# Detecting Novel and Emerging Drug Terms Using Natural Language Processing: A Social Media Corpus Study

**DOI:** 10.2196/publichealth.7726

**Published:** 2018-01-08

**Authors:** Sean S Simpson, Nikki Adams, Claudia M Brugman, Thomas J Conners

**Affiliations:** ^1^ Georgetown University Washington, DC United States; ^2^ Center for Advanced Study of Language University of Maryland College Park, MD United States

**Keywords:** natural language processing, street drugs, social media, vocabulary

## Abstract

**Background:**

With the rapid development of new psychoactive substances (NPS) and changes in the use of more traditional drugs, it is increasingly difficult for researchers and public health practitioners to keep up with emerging drugs and drug terms. Substance use surveys and diagnostic tools need to be able to ask about substances using the terms that drug users themselves are likely to be using. Analyses of social media may offer new ways for researchers to uncover and track changes in drug terms in near real time. This study describes the initial results from an innovative collaboration between substance use epidemiologists and linguistic scientists employing techniques from the field of natural language processing to examine drug-related terms in a sample of tweets from the United States.

**Objective:**

The objective of this study was to assess the feasibility of using distributed word-vector embeddings trained on social media data to uncover previously unknown (to researchers) drug terms.

**Methods:**

In this pilot study, we trained a continuous bag of words (CBOW) model of distributed word-vector embeddings on a Twitter dataset collected during July 2016 (roughly 884.2 million tokens). We queried the trained word embeddings for terms with high cosine similarity (a proxy for semantic relatedness) to well-known slang terms for marijuana to produce a list of candidate terms likely to function as slang terms for this substance. This candidate list was then compared with an expert-generated list of marijuana terms to assess the accuracy and efficacy of using word-vector embeddings to search for novel drug terminology.

**Results:**

The method described here produced a list of 200 candidate terms for the target substance (marijuana). Of these 200 candidates, 115 were determined to in fact relate to marijuana (65 terms for the substance itself, 50 terms related to paraphernalia). This included 30 terms which were used to refer to the target substance in the corpus yet did not appear on the expert-generated list and were therefore considered to be successful cases of uncovering novel drug terminology. Several of these novel terms appear to have been introduced as recently as 1 or 2 months before the corpus time slice used to train the word embeddings.

**Conclusions:**

Though the precision of the method described here is low enough as to still necessitate human review of any candidate term lists generated in such a manner, the fact that this process was able to detect 30 novel terms for the target substance based only on one month’s worth of Twitter data is highly promising. We see this pilot study as an important proof of concept and a first step toward producing a fully automated drug term discovery system capable of tracking emerging NPS terms in real time.

## Introduction

With the rapid development of new psychoactive substances (NPS) and changes in the use of more traditional drugs, it is increasingly difficult for researchers and public health practitioners to keep up with emerging trends in how these substances are referred to by their users. Further complicating the matter, linguistic innovation is often magnified with the discussion of taboo substances and behaviors, which may cause drug-related vocabulary to emerge and recede more quickly than vocabulary in other domains [1]. Developing a method for detecting and tracking emerging drug terms in near real time is essential for researchers and professionals working in drug-related fields, yet most Web-based compendia of drug terms put out by official agencies such as the Centers for Disease Control and Prevention (CDC) and the Drug Enforcement Agency (DEA) are infrequently updated and rarely contain the most recent terms.

Social media use offers an opportunity to address this problem, since these communications can be one of the earliest records of innovative use of vocabulary [2]. Furthermore, streaming social media corpora can be continuously updated as new posts are uploaded, ensuring that such corpora always reflect language use on the platform in near real time.

This article details a pilot study designed to explore the feasibility of applying methods for synonym detection drawn from the field of natural language processing to a streaming social media corpus comprising posts (tweets) to the microblogging platform Twitter in order to uncover novel terms referring to marijuana. Though marijuana was used as the target substance here, the goal of this pilot study was to develop a method for drug-term discovery that may be extended to other illicit substances and other social media platforms. It is our hope that this line of research will eventually lead to a system that is easily deployable, capable of providing public health practitioners with lists of slang and street terms currently in use for most illicit drugs of interest, and updated continuously in near real time.

### Social Media and Health

Though it has not to our knowledge been used for the purpose of uncovering new drug terms, the use of social media corpora in the discovery and analysis of drug-related health information more generally is not a novel concept. For example, Paul and Drezde [3] showed that it is possible to use a social media corpus to automatically learn relationships between drugs, their routes of administration, and other aspects of use. Recent years have also seen a rush of new work in the area of pharmacovigilance, where social media has been used to monitor adverse drug reactions (see [4] for a recent overview of research on the utility of social media monitoring for pharmacovigilance). Sinnenberg et al [5] provide an overview of the use of Twitter specifically for health care research, including drug-related applications. However, the majority of previous studies employing social media to investigate drug-related health trends (eg, [6-9]) have typically either used relatively small-scale datasets or have specifically precluded attention to unknown terms for the drug and therefore ruled out new term discovery [10].

### Synonym Detection

The task of uncovering novel terms that refer to known drug substances may in many respects be boiled down to an exercise in emergent synonym detection. In other words, in order to find new terms that refer to known drugs, an obvious way to go about doing so is to search for new terms that are synonymous or nearly synonymous with *known* terms for said drugs.

Synonym detection has been a richly explored area in information retrieval, and over the last decade, it has become a subject of increasing interest in various fields of health science and medicine. Most modern approaches employ some species of vector space model (VSM; sometimes referred to as a vector space model), in which words within a corpus are represented as continuous vectors (also called word embeddings) in high-dimensional vector space. These word vectors are constructed with respect to the linguistic contexts in which they occur within a given corpus, meaning that words appearing in similar contexts (ie, with similar surrounding words) will have similar vector representations. This feature of VSMs allows them to take advantage of the distributional hypothesis of semantics—the idea that words occurring in similar linguistic contexts tend to have similar meanings, and conversely that words with similar meanings tend to occur in similar contexts [11,12]. Insofar as the vector representation of a word accurately captures the probabilistic linguistic contexts in which it is likely to appear, the spatial similarity (measured as cosine similarity between vector angles within the *n*-dimensional vector space) between any 2 word vectors within the model can be taken as a proxy measure for the degree of semantic similarity between the 2 target words.

VSMs have been used within health science and medical research for a number of tasks related to synonym detection. Kuffner et al [13], for example, describe a vector space approach termed ConceptMaker to detect associational relations between gene and protein names in the NCBI PubMed corpus. Similarly, Henriksson et al [14] use a combination of vector space methods known as random indexing (RI) [15] and random permutation (RP) [16] to uncover and map the relation between different medical terms that are used synonymously across different clinical contexts. Henriksson et al [17] use a similar ensemble of RI and RP to uncover synonymic relations between various idiosyncratic abbreviations and their expansions within a medical context.

Despite the interest in semantic-relatedness detection and the increasing use of VSMs to achieve this goal in areas related to public health, we are unaware of any attempts so far to apply this type of approach to the problem of emergent drug terms. It is the hope of the researchers that by applying such an approach to a continually updating social media corpus, it will be possible to create a system capable of uncovering and tracking novel and emerging drug terms in near real time.

## Methods

Broadly, the method employed here can be summarized into four steps: (1) a VSM was trained over a large Twitter corpus to map all the terms within the corpus with respect to semantic similarity, (2) in consultation with drug-research experts, 2 well-known and prevalent street terms were selected to serve as the target query terms for the target substance—that is, the prototypical street terms for the target substance to which other such street terms within the corpus should hold a high degree of semantic similarity, (3) the word vectors within the trained VSM were sorted according to semantic similarity to the target query terms and filtered to exclude any terms below an optimized threshold for semantic similarity, creating a candidate list of those terms within the corpus which could be considered as most likely to refer to the target substance, and (4) this candidate term list was then evaluated by hand to determine which of the candidate terms in fact referred to the target substance and which terms were false positives. These steps are presented in more detail in the following subsections.

### Data Collection and Preprocessing

This study was conducted using a corpus of Twitter messages collected by the National Drug Early Warning System (NDEWS) Coordinating Center at the Center for Substance Abuse Research (CESAR). The corpus comprises tweets continuously collected from a *spritzer* level (ie, a random 1% sampling of all tweets) connection to Twitter’s streaming application programming interface (API) from October 1, 2015 until the present. The incoming tweet stream was filtered to exclude tweets written in languages other than English and those originating from outside the continental United States of America. Due to these requirements, tweets with no associated geographic or language metadata were excluded. For this pilot study, we used a subset of this larger Twitter corpus, comprising tweets from July 1 to July 31, 2016. Before analysis, the subset was preprocessed to remove Twitter handles (eg, @MyCoolName) and URL links. Hashtags (eg, #NLProc), and emoticons or emojis (eg, :) or 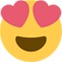) were retained. All tweets were then tokenized using the tweet tokenizer from the Natural Language Tool Kit (NLTK) Python package [18]. The resulting dataset contained 82.6 million tweets and 884.2 million tokens.

It is worth noting here that focusing only on geotagged tweets when analyzing Twitter data inherently introduces a sampling bias, in that doing so samples only from those users who choose to turn on geolocation services—a subpopulation that may not be representative of Twitter users as a whole [19]. However, because we are specifically interested in drug terms as used in the United States, ensuring that all tweets come from the target population was deemed more important for this study than avoiding such a sampling bias.

### Selection of Target Substances and Terms

For this pilot study, marijuana was chosen as the substance of primary focus because of its relatively high level of use within the American population [20] and comparatively low social stigmatization [21,22]. Frequent, casual discussion of this substance over Twitter was hypothesized to accord us enough linguistic data to assess the efficacy of the current method under circumstances of high volume and high noise. In consultation with our drug-research expert collaborators, 2 slang terms were selected for subsequent model querying based on the frequency in the corpus and geographic ubiquity of use: *weed* and *ganja*. These terms served as the prototypical street terms for marijuana to which other such street terms in the corpus were hypothesized to have a high degree of semantic similarity during candidate term retrieval.

### Modeling

Before training the VSM, the corpus was analyzed in order to identify 2-word sequences (bigrams) that had an unusually high probability of appearing together as a unit and were therefore likely better treated as one multiword token rather than 2 separate tokens (eg, sequences of *los angeles* were treated as one token *los_angeles* rather than 2 separate tokens *los* and *angeles*). Multiword token identification was accomplished using the method described by Mikolov et al [23], implemented in the open-source software package *gensim* [24]. The resulting corpus was then used to train a continuous bag of words (CBOW) VSM of the type introduced by Mikolov et al [25], again implemented in *gensim*. After hyperparameter tuning, our final model was trained with a context window of 5 and a dimensionality of 200, excluding from the vocabulary tree any terms that did not appear at least 10 times within the training corpus. The resulting VSM included 662,742 unique token-type word vectors.

### Candidate Term Retrieval

Recall that the spatial similarity between 2 word vectors (measured in cosine similarity—that is, the cosine of the angle between 2 word vectors) can be taken as a proxy for semantic similarity in trained VSMs. Therefore, to extract from the trained model a list of terms that were most likely to refer to the target substance, we first calculated the cosine similarity between each of the 662,742 word vectors within the trained model and our target query terms, with the assumption that those token-types with the highest cosine similarity to our query terms would in turn have the highest degree of semantic similarity and thus have the highest likelihood of referring to the target substance. Having done so, we were then able to sort the corpus in descending order of semantic similarity to our 2 target query terms. To pare down the list of terms and reduce the number of false positives, it was necessary to select a cosine similarity threshold below which terms were considered too dissimilar from our target query terms to be likely to refer to our target substance. An optimum threshold would result in a list that includes as few false positives as possible, yet includes most or all terms used in the corpus to refer to the target substance. In other words, an optimal threshold for this particular task is one which results in a relatively high recall rate (ie, percentage of all possible terms for the target-substance that were included in the resulting candidate list) and an acceptable level of precision (ie, percentage of terms on the resulting candidate list which in fact refer to the target substance) such that a human reviewer with expert knowledge could vet all the terms on the list in a matter of hours. An obvious problem in calculating recall and precision in this context, however, is that the true status of any of the terms in the corpus with respect to whether or not they refer to the target substance is unknown. Therefore, we reached out to community researchers at three of the 12 regional drug research sites of the NDEWS. These three sites (Denver, Chicago, and Philadelphia) exist in communities experiencing significant substance abuse or misuse problems [26]. The field experts were asked to provide a list of terms for marijuana that they considered to be currently in use in their own communities. Collating the lists obtained from each of these three groups of field experts resulted in a list of 32 unique terms, including our 2 target terms. These 32 terms and common spelling variants thereof were then considered as the only terms that referred to the target substance for the purpose of cosine similarity threshold optimization.

**Figure 1:**
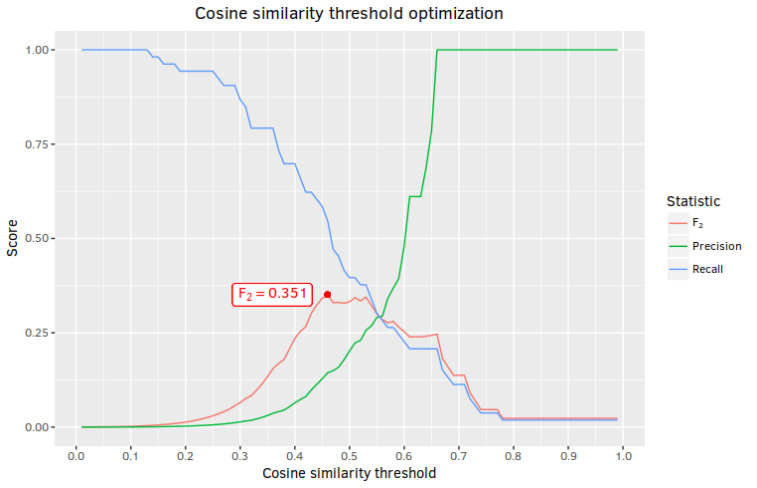
Recall, precision and F2 scores for the ganja optimization run.

With the expert generated list in hand, we calculated recall and precision for each possible cosine similarity threshold from 0.01 to 1.00 in steps of 0.01, optimizing for an F-measure with a beta value of 2 (F_2_ rather than F_1_ was chosen as our optimization function because we place more importance on recall than precision for this task). To determine which query term or combination of query terms resulted in the highest recall, precision, and F_2_ score, optimization runs were performed under four conditions: once for each of the 2 target query terms using only the cosine similarity results for that query term, once using the set union of cosine similarity results for both query terms (ie, all terms in the corpus with a level of cosine similarity above the given threshold for *either* term), and once using the cosine similarity results calculated against the simple mean of the 2 target query vectors. Recall, precision, and F_2_ values for the cosine similarity threshold at which maximum F_2_ was achieved for each of the four runs is presented in Table 1. F_2_, recall, and precision for all thresholds during the optimization run with the highest resulting F_2_ score are presented in Figure 1.

As the candidate term list created using only *ganja* as the prototypical query vector with a cosine similarity threshold of 0.46 achieved the highest F_2_ scores of all four runs, this was the candidate term list used moving forward. Considering all terms with a cosine similarity of 0.46 or greater to our target query term *ganja* resulted in 200 terms in total (This number is somewhat inflated because spelling variants are treated as unique items. For example, *weed* and *weeed* are counted as distinct terms in this list.).

**Table 1:** Cosine similarity threshold optimization.

Term or unit	Cosine similarity threshold	F_2_	Recall	Precision
ganja	0.46	0.351	0.547	0.144
weed	0.49	0.280	0.453	0.111
union	0.53	0.295	0.396	0.146
simple mean	0.50	0.306	0.490	0.122

**Table 2:** Candidate term categories.

Category	Description	Examples
Marijuana	Terms for the substance itself, including specific strain names	kush dro sour diesel
Paraphernalia or process	Marijuana related, but not terms for the substance itself	doobie blunts bong rips
Other-drug	Drug related but not marijuana-related	opium shrooms mdma
Nondrug	Unrelated to drugs specifically	rasta catnip herbal

### Candidate Term Categorization

Though only terms in the expert-generated list were treated as marijuana-related for the purpose of cosine similarity threshold and query term optimization, the major goal of this study was to determine whether the current method captures terms for the target substance that are unknown to such experts. To do so, all terms on the resulting candidate term list were then classified by 2 researchers into the following four categories: marijuana, process or paraphernalia, other-drug, and nondrug. A description of these categories along with examples is given in Table 2.

First-pass categorization was performed using lists of possible slang terms for marijuana and associated processes and paraphernalia published by the DEA [27] and the Center for Substance Abuse Research (CESAR) [28]. All candidate terms included on these lists were categorized as either (i) *marijuana* or (ii) *process* or *paraphernalia*. Second-pass categorization consisted of searching websites such as Urban Dictionary [29] and Marijuana Dictionary [30] as well as marijuana-focused online drug forums for definitions and/or exemplar usage of the remaining terms. Candidate terms for which the researchers could determine that the term referred to marijuana were classified as *marijuana*. Terms determined to refer to ingesting marijuana or to tools used in ingesting marijuana were categorized as *process* or *paraphernalia*. Terms that we were able to confirm as relating to the drug realm but to a substance other than marijuana were categorized as *other-drug*. Terms for which we were unable to find any sort of drug-related meaning or usage or terms for which the exact meaning was unclear were categorized as *nondrug*. Both researchers categorized the candidate terms independently, agreeing in 78.0% (156/200) of cases (Cohen kappa=.703). A third researcher evaluated cases in which the primary categorizers disagreed. These cases were categorized according to the judgment of the third categorizer (there were no cases in which the third categorizer did not agree with either of the primary categorizers).

### Evaluation Metrics

The overarching goal of this study was to develop a method capable of mining social media to produce a list of candidate terms that are highly likely to refer to a target substance (in the case of this pilot study, marijuana). Success in this task is characterized by producing a candidate term list with the following three characteristics:

1. Includes all terms for the target substance known to experts in the field
2. Includes terms for the target substance *not* known to experts in the field
3. Includes a minimal number of false positives

To determine success with respect to the first criterion, we determine the recall for our candidate term list with respect to marijuana terms on the term list obtained from the field experts at NDEWS. To determine success with respect to the second criterion, we removed those terms which appeared on the expert-derived list from the set of terms which during categorization were determined to be terms for the substance itself. Those terms which were determined to be used to refer to the substance itself yet which were not included on the expert-generated lists were deemed to be novel for our purposes and possibly unknown to researchers. To determine success with respect to the third criterion, we evaluated the precision of the resulting candidate list treating terms categorized as *marijuana* as true positives and all other terms as false positives.

## Results

Table 3 summarizes the category frequency for terms in the candidate term list as determined by the categorization procedure described above.

Of the 200 terms returned as potential terms for marijuana, 86.0% (172/200) were drug-related. Of the drug-related terms, 115 (57.5% of all terms) were marijuana-related and 65 (32.5% of all terms) referred to the substance itself. A list of the candidate terms classified as *marijuana* is provided in Table 4. The third column provides the cosine similarity within our VSM between the listed term and the query term *ganja*.

Some terms in Table 4 are well known (eg, *reefer* and *weed*), and some are orthographic variants of one another (eg, *weed* and *weeed*). Several, such as *gorilla glue* or *hawaiian punch*, refer to specific strains of marijuana. Researching these terms on the various drug forum websites [29,30] suggests that some may be relatively new or are gaining popularity within marijuana-focused communities. The terms *moonrock* and *moonrocks,* for example, refer to a particular preparation of marijuana, apparently introduced sometime in 2012. The term *jazz cabbage* appears to have entered into marijuana parlance as recently as early to mid-2016 [29]. Some even appear to be specific to certain communities of practice, such as *pacc*: an orthographic variant of *pack*, referring to *loud pack* or good-quality marijuana. *Pacc* avoids the *ck* letter combination that is taboo among members of the Crip gang (as it can represent *Crip killer*) [31]. Interestingly, one of the candidate terms used to refer to marijuana is not traditionally thought of as a *term* at all, but rather a sequence of 2 *leaf fluttering in wind* emoji characters. The use of leaf-based emojis is mentioned as an obfuscatory tactic to covertly reference marijuana in a number of online drug-focused blogs, such as [32], but so far as we are aware is not acknowledged in any of the lists of marijuana-signifying terms put out by the CDC or other official organizations.

### Evaluation Outcomes

As mentioned, we consider 3 criteria in evaluating the candidate term list produced via the above method: 1) recall with respect to terms on the expert-generated list, 2) number of terms determined to refer to marijuana but not appearing on any of the expert-generated lists, and 3) precision with respect to terms relating to the substance of marijuana.

#### Comparison of Candidate List to Expert-Generated Lists

Of the 35 terms for marijuana provided by NDEWS experts, 91%, or all but 3 terms— *sour d*, *blue cheese*, and *love boat* —occurred within the corpus. These 3 terms were excluded from further analysis.

The remaining 32 terms from the expert-provided list are given in Table 5. As with Table 4, for each row, column 3 provides the cosine similarities between the listed term and the target query term *ganja*. For each term we wanted to know not only whether it was included at all within the corpus but also whether it was used in the expected, drug-related sense—and if so, how often. To capture this, column 6 of Table 5 presents a measure of drug-relevancy for each term—that is, the percentage of instances in which that term was used to refer to the target substance.

Drug-relevancy was calculated as follows. For each term in the list of 32, a random sample of 200 tweets containing that term was extracted from the corpus (for cases in which the term appeared fewer than 200 times in the corpus, all instances were extracted.). Each tweet was then coded independently by 2 researchers to determine whether the term referred to the target substance or not. These annotators were highly consistent with one another in terms of the percentage of tweets determined to refer to the target substance for each term (r=.978). The average between the 2 annotators for the percentage of tweets in which the term was judged to be used to refer to the target substance was then taken as a proxy for the overall *drug-relevancy* of that term within the corpus. If the term was not used to refer to the target substance in any of the randomly sampled tweets that contained it, it received a drug-relevancy of 0%. If the term was used to refer to the target substance in all of the sampled tweets, it received a drug relevancy of 100%.

The terms in Table 5 are sorted by descending drug-relevancy. Those items marked with a superscript a represent terms provided by NDEWS experts which were included in the model-derived list of 200 candidate terms. Those terms marked with a superscript b represent expert-provided terms that were not included in the candidate term list returned by our model queries. Columns 4 and 5 reflect the number of tweets judged to refer to the target substance out of the total number of tweets sampled for each annotator. Column 6 represents the average of drug-relevancy ratings between the 2 annotators.

**Table 3:** Category frequency of candidate terms.

Category	Number	Percentage
Marijuana	65	32.5
Process or paraphernalia	50	25.0
Other-drug	57	28.5
Nondrug	28	14.0
Total	200	100

**Table 4:** Marijuana terms from candidate list.

Rank	Term	Cosine similarity to query term ganja
1	kush	0.772
2	reefer	0.738
3	dro	0.722
4	reefa	0.717
5	weed	0.712
6	sativa	0.682
7	sour diesel	0.676
8	purp	0.670
9	devils lettuce	0.670
10	og kush	0.667
11	doja	0.654
12	kief	0.649
13	gorilla glue	0.640
14	thrax	0.633
15	piff	0.632
16	weeed	0.605
17	moonrocks	0.603
18	pacc	0.603
19	tookah	0.601
20	devil's lettuce	0.600
21	moonrock	0.598
22	tooka	0.587
23	edibles	0.584
24	mids	0.581
25	bubba kush	0.563
26	gasss	0.562
27	gass	0.562
28	marijuana	0.556
29	ganj	0.550
30	dodi	0.546
31	indica	0.544
32	jazz cabbage	0.543
33	faygo	0.540
34	dank	0.539
35	dabs	0.538
36	oregano	0.533
37	wata	0.527
38	bammer	0.527
39	tincture	0.517
40	marijuanas	0.514
41	k2	0.513
42	thc	0.497
43	gas	0.495
44	rosin	0.493
45	smarties	0.492
46	pot	0.490
47	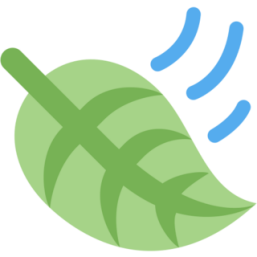 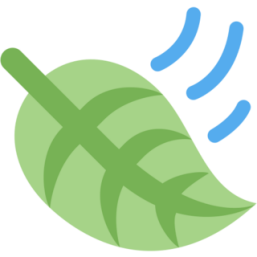	0.489
48	gassss	0.488
49	danky	0.487
50	hemp	0.483
51	weeeeed	0.480
52	herb	0.477
53	kool aid	0.473
54	hawaiian punch	0.472
55	cannabis	0.469
56	reggie	0.469
57	jolly ranchers	0.468
58	kushy	0.465
59	grape juice	0.464
60	cheech	0.461
61	goop	0.461
62	khalifa kush	0.461
63	tropical fusion	0.461
64	broccoli	0.460
65	medicinal	0.460

In total, 15 out of the 32 terms present in the expert-derived term lists appeared on our candidate term list. This translates to a recall rate of 46.9%. Although at first blush, this appears to be relatively low, this is quite similar to recall rates that others have obtained in related tasks. Henriksson et al [17], for example, report a recall rate of between 33% and 47% using a similar method in performing synonym and abbreviation detection in medical texts. Similarly, Henriksson et al [14] report a recall rate of 44% in matching synonymous medical terms across different genres of clinical text.

Sorting Table 5 by descending drug relevancy appears to provide some insight as to why our method resulted in the inclusion of some of the expert terms on our candidate term list but not others. Every term on the expert-derived list with a drug relevancy of 21.8% or higher was included in our candidate list (items marked with superscript a in Table 5), whereas every expert-derived term with a drug relevancy of less than 21.8% was not (items marked with superscript b in Table 5). This suggests that while recall over the whole expert-derived list was somewhat low, our method actually performed quite well above a certain relevancy threshold—namely when the term was used to refer to the target substance in at least roughly one out of every five cases within the corpus. That is to say, though recall was 46.9% overall, examining the drug relevancy of each term reveals that recall for terms with at least 21.8% relevancy was actually 100%, whereas recall for terms with below this relevancy threshold was 0%.

#### Discovery of Novel Terms

In addition to uncovering all marijuana-related terms on the expert-generated list with a drug relevancy of at least 21.8%, our model also returned a number of marijuana-related terms that were not included on the lists provided by experts. In all, 65 of the 200 terms on the candidate term list were determined to refer to the target substance itself. Of these, 29 could be considered known terms for our purposes—that is, either technical terms of which we can assume experts to be aware (eg, *thc* and *cannabis*) but which were not included on the expert-generated list (as experts were explicitly instructed to include slang and street terms, not technical terms), or terms specifically included on the expert-generated lists (eg, *kush*) and spelling variants thereof (eg, *kushy*). Excluding spelling variants, the remaining 36 terms accounted for 23 unique terms for the general substance marijuana (eg, *thrax* and *piff*) and 7 unique terms for specific strains of marijuana (eg, *gorilla glue* and *hawaiian punch*). These 30 terms, which we consider for our purposes to be novel (ie, not included on the expert-generated list), are provided below in Table 6.

**Table 5:** Expert-generated marijuana terms.

Rank	Term	Cosine similarity	Drug relevancy		
			Annotator 1	Annotator 2	Average relevancy, %
1	edibles^a^	0.58	180/200	185/200	91.3
2	weed^a^	0.71	186/200	177/200	90.8
3	ganja^a^	1.00	190/200	169/200	89.8
4	kush^a^	0.77	192/200	165/200	89.3
5	sativa^a^	0.68	176/200	177/200	88.3
6	sour diesel^a^	0.68	91/112	98/112	84.4
7	indica^a^	0.54	132/200	170/200	75.5
8	devil’s lettuce^a^	0.67	145/200	150/200	73.8
9	dro^a^	0.72	136/200	134/200	67.5
10	dabs^a^	0.54	123/200	142/200	66.3
11	purp^a^	0.67	75/200	59/200	33.5
12	pot^a^	0.49	64/200	69/200	33.3
13	dank^a^	0.54	56/200	43/200	24.8
14	herb^a^	0.48	37/200	54/200	22.8
15	reggie^a^	0.47	41/200	46/200	21.8
16	wax^b^	0.45	42/200	43/200	21.3
17	nug^b^	0.36	16/200	55/200	17.8
18	mary jane^b^	0.31	24/200	36/200	15.0
19	pineapple express^b^	0.38	29/200	27/200	14.0
20	chronic^b^	0.26	35/200	18/200	13.3
21	shatter^b^	0.16	18/200	32/200	12.5
22	bud^b^	0.36	20/200	21/200	10.3
23	skunk^b^	0.31	24/200	14/200	9.5
24	haze^b^	0.36	13/200	17/200	7.5
25	exotics^b^	0.19	9/143	12/143	7.3
26	hydro^b^	0.32	15/200	12/200	6.8
27	shard^b^	0.14	5/138	4/138	3.3
28	flower^b^	0.29	1/200	9/200	2.5
29	fire^b^	0.30	0/200	10/200	2.5
30	mud^b^	0.31	1/200	4/200	1.3
31	flame^b^	0.40	0/200	5/200	1.3
32	green^b^	0.25	1/200	3/200	1.0

^a^Terms provided by author-affiliated experts included in the model-derived list of 200 candidate terms.

^b^Expert-provided terms that were not included in the candidate term list returned by our model queries.

**Table 6:** Marijuana terms on candidate list but not on expert lists.

Number	Term	Note
1	bammer	
2	broccoli	
3	cheech	
4	dodi	
5	doja	
6	gas or gass or gasss or gassss	
7	goop	
8	jazz cabbage	
9	kief	
10	mids	
11	oregano	
12	pacc	
13	piff	
14	reefer or reefa	
15	thrax	
16	tooka or tookah	
17	moonrock or moonrocks	particular preparation
18	rosin	particular preparation
19	tincture	particular preparation
20	wata	particular preparation
21	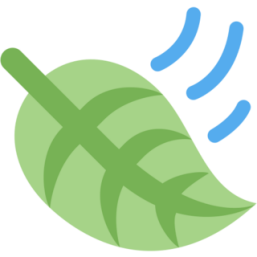 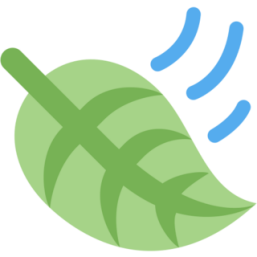	*leaf in wind* emoji sequence
22	k2	synthetic marijuana
23	faygo	strain
24	gorilla_glue	strain
25	grape_juice	strain
26	hawaiian_punch	strain
27	jolly_ranchers	strain
28	kool_aid	strain
29	smarties	strain
30	tropical_fusion	strain

Some of those terms included in Table 6 such as *reefer* are well known in general and surely known to experts, despite the fact that they were left off of the expert-generated lists used here. However, several of the general terms listed in Table 5 as well as strain names appear to be relatively new and thus perhaps truly unknown to many experts in the field. Regardless, the large number of terms uncovered by the current method which were not part of the expert-generated lists suggests that this may be a fruitful method for detecting drug terms of which drug-research experts may not yet be aware.

### Precision of the Current Method

In total, 65 of the 200 terms included on the candidate term list were determined to refer to the substance marijuana, resulting in a precision of 32.5%. This means that as it currently stands, a human is still needed to review candidate lists produced by the method evaluated here before distributing any such lists to public health practitioners—a less than desirable outcome. However, at this stage no attempts have yet been made to post process the candidate list in an attempt to weed out the false positives. There are several relatively simple methods of doing so, which would raise the precision rate, such as eliminating from the candidate list all terms that are not generally used as nouns (thereby eliminating from the list false positives such as *smoking*), using a stop-list to exclude common terms for known drugs that are not the target drug (thereby eliminating false positives such as *cocaine* and *mdma*) and so on. This is an area for future work and refinement.

## Discussion

### Principal Findings

On the whole, we consider this pilot study to be an important proof of concept. Though the candidate term list had a relatively low recall rate with respect to the expert-generated terms overall, annotating the expert terms for drug relevancy revealed that the terms that were not in the candidate list were wholly predictable. All expert-derived terms that were used in the corpus to refer to the target substance in 21.8% or more of instances were included on the candidate list, whereas all expert-derived terms with relevancy rates lower than 21.8% were not. This suggests that whereas future work should focus on methods of improving identification of low drug-relevancy terms, our method performs quite well in capturing terms with a drug relevancy over this (relatively low) threshold.

In addition, our method enabled us to identify 30 novel terms for the target substance which were not included on any expert-derived list, nearly equaling the number of terms provided on the expert list in the first place. On the basis of the recall rate with respect to the expert terms, it seems likely that our candidate list includes all or most terms used in the corpus to refer to the target substance in at least 21.8% of instances, though of course this is impossible to know for certain. These are encouraging results and suggest that the method described above and subsequent refinements thereof can be a viable framework for the detection of novel drug terms using social media.

### Strengths and Limitations

An obvious shortcoming of this method is that it performs poorly in identifying terms that have drug-relevant meanings, but which are only used in drug-relevant senses in fewer than 22% of cases. This is a significant shortfall, as taboo terms are often reappropriations of existing words into new meanings [1]. It is unclear at the moment as to how to go about addressing this issue. One possible solution is to require a step that takes advantage of other grammatical and semantic information to disambiguate homographs either before or concurrent with training the VSM. One such word sense disambiguation method introduced in late 2015 is sense2vec [33]. The recently introduced Word to Gaussian Mixture (w2gm) model framework [34], which models each word as a mixture of Gaussians, may also prove useful for the purpose of sense disambiguation. Both of these approaches will be explored in future work.

A further limitation of this method is the relatively low precision rate (32.5%) of the model-generated candidate term list with respect to terms referring to the target substance. Whereas, at present, this precludes the possibility of eliminating human review of the candidate term list before distributing it to health professionals, there are various methods for postprocessing, which may substantially raise the precision rate, and that have yet to be explored. It is also possible that training word embeddings on a longer time slice of the corpus (ie, more data) would result in higher quality word embeddings, potentially raising rates of both precision and recall. These are areas of ongoing work.

Finally, although this pilot study has been reasonably successful and has demonstrated proof of concept, the extensibility of the method used here for detecting drug terms referring to substances other than marijuana is unclear. Trial runs targeting terms for methamphetamine and heroin suggest that these drugs are not discussed as frequently on Twitter and therefore appear potentially unsuitable for this sort of analysis. Initial inquiry into the application of this method to prescription drugs such as oxycontin and *party* drugs such as MDMA, however, appears encouraging.

### Future Work

This pilot study made use of a 1-month portion of the Twitter corpus, thereby giving us a static snapshot of language use on Twitter at that time. An area of future research is to perform a trend study, looking at the way language use surrounding a particular target substance changes over time. Such a trend study could provide insight on the rate of rise and fall of drug vocabulary, as well as how terms spread geographically throughout the country.

Relatedly, taking advantage of the geotag metadata associated with the tweets collected could provide important dialectal data. The model employed for this study is a national model, however, the same method could be applied to tweets binned by geotag. In theory, this could reveal the use of different terms in different regions, though success may be mitigated by the necessarily smaller volume of data circumscribed in that way.

Extensions of this method to different areas of social media are also warranted. It may be that discovery of novel terms for more socially stigmatized substances may require the use of corpora from platforms that lend themselves more to user anonymity. Applications of the method developed above using corpora drawn from the discussion forum website Reddit, as well as from several online forums specifically geared toward drug use are currently being planned.

As our focus here has been on uncovering terms specifically referring to the target substance, we should note that we have not explored in detail why terms for certain nontarget drugs appear in the candidate list in addition to terms for the target substance. It may be that we are collecting terms related to drugs of a certain category or drugs that have similar effects and are therefore talked about similarly in the corpus. Thus, our candidate list may reflect a broader conceptual category on the part of users, such as *party drug*. If this is the case, attention in this area may lead to the development of a model representing users’ knowledge of drug behaviors, which may in turn reveal novel practices such as new combinations of drugs.

These topics are currently being explored by the research team in collaboration with the NDEWS Coordinating Center.

### Conclusions

Twitter represents a fruitful venue in which to identify and track emerging drug term trends, particularly with reference to terms for marijuana. Furthermore, the VSM model and approach documented here successfully identified new terms heretofore unknown to many experts in the field.

## Abbreviations

API: application programming interface
CESAR: Center for Substance Abuse Research
CBOW: continuous bag of words
CDC: Centers for Disease Control and Prevention
DEA: Drug Enforcement Agency
NPS: new psychoactive substances
NLTK: natural language tool kit
RI: random indexing
RP: random permutation
VSM: vector space model
